# Unlocking fertility in the female gametophyte: a DEAD-box RNA helicase is essential for embryo sac development and seed setting

**DOI:** 10.1093/jxb/erae220

**Published:** 2024-08-22

**Authors:** Asif Ali, Asad Riaz, Xianjun Wu

**Affiliations:** State Key Laboratory of Crop Gene Exploration and Utilization in Southwest China, Rice Research Institute, Sichuan Agricultural University, Chengdu, 611130, China; Centre of Excellence for plant success in nature and agriculture, The Queensland Alliance for Agriculture and Food Innovation (QAAFI), The University of Queensland, St Lucia, Brisbane, QLD 4067, Australia; State Key Laboratory of Crop Gene Exploration and Utilization in Southwest China, Rice Research Institute, Sichuan Agricultural University, Chengdu, 611130, China

**Keywords:** DEAD-box helicase, degeneration, embryo sac development, female gametophyte, fertility, seed setting rate

## Abstract

This article comments on:

**Huang J, Qiao Z, Yu H, Lu Z, Chen W, Lu J, Wu J, Bao Y, Shahid MQ, Liu X.** 2024. OsRH52A, a DEAD-box protein, regulates functional megaspore specification and is required for embryo sac development in rice. Journal of Experimental Botany **75**, 4802–4821. https://doi.org/10.1093/jxb/erae180

**Reproductive success and seed production rely on normal development and function of male and female organs. Previous studies reported that reproductive organs are prone to degeneration due to excessive reactive oxygen species (ROS), increased programmed cell death (PCD), developmental defects, and abnormal cell division. An embryo sac is a structure present in an ovule and is essential for developing an egg. Huang *et al.* (2024) revealed that loss of the DEAD-box RNA helicase OsRH52A in rice leads to abnormal development of the embryo sac including the appearance of double-female gametophyte structures and, as a consequence, to low seed setting.**

A flowering plant has two phases in its life cycle, diploid sporophytic generation and haploid gametophytic generation. Generally, both male and female gametophytes are present in the same plant and are essential for sexual reproduction. A female gametophyte is required to guide the pollen tube to the ovary for fertilization and induction of seed development (Yadegari and Drews, 2004). Development of the female gametophyte is a highly organized process that occurs within an ovule and has been well reviewed in terms of several aspects, such as zygote activation (Xie *et al.*, 2022), zygote embryogenesis (Dresselhaus and Jürgens, 2021), and embryo–endosperm interaction (Doll and Ingram, 2022). However, how defects of the female gametophyte lead to sterility and low yield remained elusive.

Embryo sac formation begins with megasporogenesis, where a megaspore mother cell (a diploid cell) undergoes meiosis, resulting in four haploid megaspores (Fig. 1). Typically, three of these megaspores degenerate, and the remaining one undergoes three rounds of mitotic division, leading to an eight-nucleate structure called the mature embryo sac. A mature embryo sac consists of three antipodal cells at the chalazal end (opposite the egg cell), two synergids and an egg cell near the micropylar end (closest to the opening for pollen tube entry), and two polar nuclei in the central cell. The fusion of the central cell (2*n*) with a sperm cell (*n*) during double fertilization forms the triploid endosperm, which provides nourishment for the developing embryo. The formation of an egg apparatus, comprising an egg cell and two synergids, is essential for fertilization. The antipodal cells, whose function is not entirely clear, are believed to play a role in nutrient transfer or hormone production. This intricate process ensures successful sexual reproduction in angiosperms by preparing the female gametophyte for fusion of the egg cell with the male gamete (sperm cell).

**Fig. 1.**
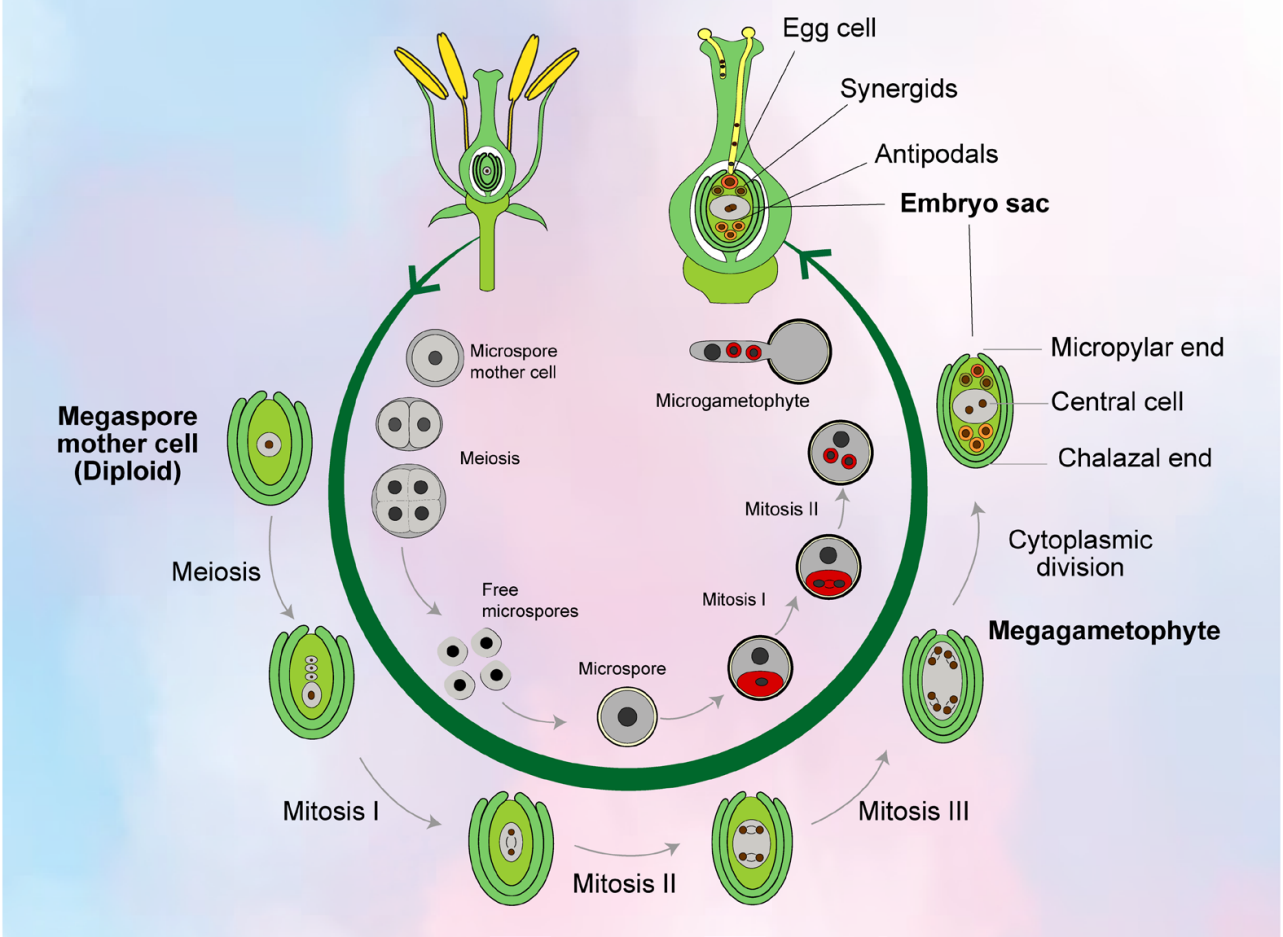
Male and female gametophyte development in flowering plants (angiosperm); see text for details.

Male and female gametes are prone to degeneration, also known as abortion, due to several factors, for example programmed cell death (PCD), excessive reactive oxygen species (ROS), lack of proper nutrition, abnormal mitosis and meiosis, and extreme environmental conditions (Yadegari and Drews, 2004; Ali *et al.*, 2019; Sankaranarayanan *et al.*, 2020). Similarly, cytological aberrations of chromosomes, cytoskeleton organization, spindle assembly, and an abnormal number of sporocytes result in the failure of normal gamete development (Zhao *et al.*, 2008; Hafidh and Honys, 2021; Shi *et al.*, 2022; Fábián *et al.*, 2024). In a recent study, *EMBRYO SAC DEVELOPMENT 1* (*ESD1*) was found to encode a protein of an ovate family that regulates seed setting by controlling the development of functional megaspores (Wang *et al.*, 2021). Similarly, *DEFECTIVE EMBRYO SAC1* (*DES1*) that encodes a nuclear envelope membrane protein is required for embryo sac development, and its absence leads to low seed setting (Hu *et al.*, 2023). However, detailed physiological and mechanistic insights into fertility are as yet unavailable. Seed setting is susceptible to many physiological and environmental conditions, and we outline a potential mechanism in Box 1.

Box 1. **Physiological mechanism of low seed setting caused by degeneration of male and female gametophytes**

Normal development of male and female gametophytes is essential for successful fertilization and ultimately seed setting. Many physiological and molecular factors have been reported to cause the degeneration or defects in male and female germlines that produce a significant reduction in seed yield (Fig. 2; Zhou *et al.*, 2017; Ali *et al.*, 2019). Previous studies reported that anthers and spikelets are prone to abortion due to increased ROS and abnormal PCD (Ali *et al.*, 2022; Zhao *et al.*, 2023), which lead to DNA fragmentation in reproductive tissues. Moreover, defects in tapetum cell death and cell division may also produce less viable pollen and cause a decrease in seed setting rate (Yan *et al.*, 2020). Similarly, other defects, such as lack of wax and cuticle formation, and defects in the morphology of male gametes, also cause excessive loss of water from the surface of anthers, which results in sterility and reduction in seed yield (Jung *et al.*, 2006). Chromosomal aberrations, complications associated with pollen tube growth, cell division, abnormal functional megaspore, and defective embryo development also result in a reduction in seed setting due to degeneration of the embryo sac (Hafidh and Honys, 2021; Fábián *et al.*, 2024).

**Fig. 2.**
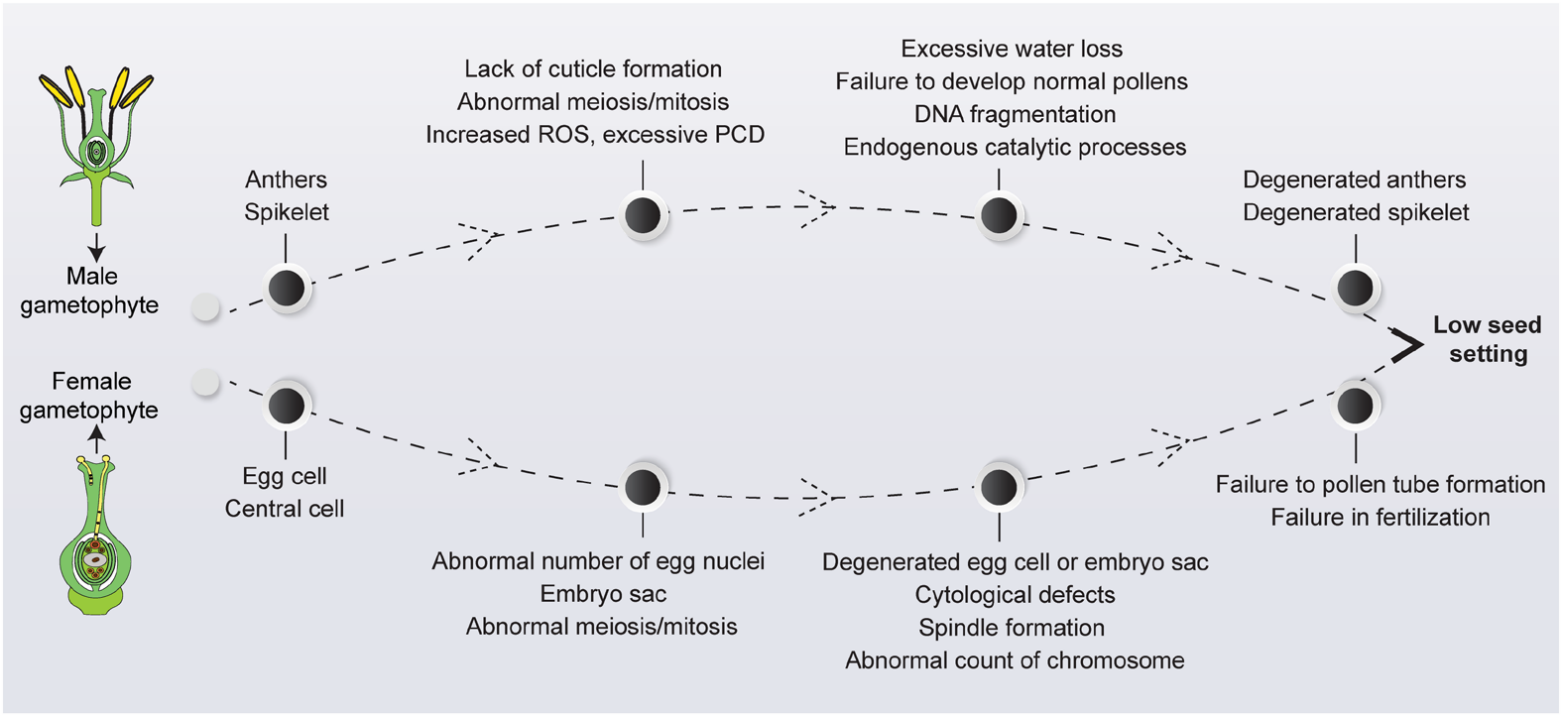
Proposed mechanism of low seed setting caused by degeneration of male and female gametophytes. ROS, reactive oxygen species; PCD, programmed cell death. See Box 1 for details.

## *OsRH52A* encodes a DEAD-box helicase and is essential for maintaining embryo sac fertility

RNA helicases of the DEAD-box family are highly conserved enzymes and are reported to perform diverse biological functions including suppression of PCD by regulating the expression of downstream genes (Linder and Jankowsky, 2011). In a previous study, Yu *et al.* (2020) discovered that a mutation in *OsRH52A* is responsible for low seed setting in neo-tetraploid rice; however, its biological function and the molecular mechanism were not explored. The expression spectrum showed that *OsRH52A* is preferentially expressed in anther and ovary development. Huang *et al.* (2024) used CRISPR/Cas9 [clustered regularly interspaced palindromic repeats (CRISPR)/CRISPR-associated protein 9] to target two sites in the genome to knock out *OsRH52A* and developed two mutants, *rh52a-m1* and *rh52a-m2*, in which nucleotide insertions or deletions led to a premature stop codon and truncated proteins lacking the DEXDc and HELICs domains which show significant reductions of up to ~35% and ~29%, respectively, in seed yield. Phenotypic analysis revealed that there were no apparent defects in the morphology of floral organs.

To investigate the reason for low seed setting, whole-mount eosin B-staining confocal laser scanning microscopy (WE-CLSM) was performed to search for defects associated with embryo sac fertility. In a recent study, Hu *et al.* (2023) also found that a mutation in *DES1*, which interacts with LONELY GUY (LOG), caused defective development of pistils and provided evidence that low seed setting in *rh52* mutants is caused by a degenerated embryo sac. Indeed, cytological observations revealed that in contrast to the wild type (WT) with only 5% abnormal embryo sacs, *rh52a-m1* and *rh52a-m2* had 35% and 41% abnormal embryo sacs, respectively (Huang *et al.*, 2024). Further analyses revealed that four different types of embryo sacs, namely the double female gametophyte (12%), degenerated embryo sacs (21%), embryo sacs without the female germline (6%), and embryo sacs without an egg and synergids (4%), were present in *rh52a-m1* and *rh52a-m2.* The results of Huang *et al.* (2024) revealed the following (but not limited to) types of aberrations in embryo sacs of *rh52a.*

(i) Double functional megaspores: under normal (WT) conditions, usually one megasporocyte divides into two cells and then into four cells following mitosis. Among these four cells, three of them undergo degeneration and only one survives, forming only one functional megaspore (Fig. 1). However, in *rh52a*, two cells were present, forming a double functional megaspore.
(ii) Double mono-nucleate embryo sacs: in the WT, one cell survives near the chalazal end; however, in *rh52a*, two cells survived, one near the chalazal end and the other near the micropylar end, and each of them developed into mono-nucleate embryo sacs forming double mono-nucleate embryo sacs.
(iii) Double bi-, tetra-, and eight-nucleate embryo sacs: in WT, the functional megaspore near the chalazal end is mono-nucleate. However, in *rh52a*, double mono-nucleate embryo sacs undergo one, two, or three mitotic cell divisions, forming bi-, tetra-, and octa-nucleate embryo sacs, respectively.

As a result of these cytological aberrations, the fertilized embryo contained two sacs (one near the chalazal end and one near the micropylar end). Among them, the one near the chalazal end could not undergo cellularization on 1 day after fertilization (DAF) and 3 DAF. Mutants defective in female gametophyte development are helpful tools for studying the complexity of sexual reproduction, as it is affected by many factors, stages, and the position of the egg cell (Yadegari and Drews, 2004; Sun *et al.*, 2021). Cytological aberrations observed in *rh52a* can be used to gain further insights into how exactly the degenerated embryo sac fails fertilization, and whether the activation, recognition, and delivery of the sperm cell were normal in *rh52a*. One possible reason for low fertility can be attributed to the fact that *rh52a* has an abnormal egg and synergids, which are essential for guiding the pollen tube. Moreover, it remains to be shown whether there are any maternal effects associated with its functional role in reproductive development and whether its overexpression can increase the seed setting. Additional studies could aim to obtain more insights into the practical implications of hybrid rice development.

## Interplay of OsRH52A with fertility-associated genes *OsMFS1* and *ZIP4*

Genetic analysis confirmed that OsRH52A is essential for functional megaspore and embryo sac development. Yeast two-hybrid (Y2H) screening was conducted to find potential interactions with other proteins involved in reproductive development. Y2H and bimolecular fluorescence complementation results revealed that OsRH52A interacts with MALE AND FEMALE STERILITY 1 (OsMFS1) and ZMM protein ZIP4. *OsMFS1* encodes a coiled-coil domain-containing protein, and dysregulation of its protein causes complete sterility in male and female organs due to abnormal meiotic recombination (Lu *et al.*, 2020). ZIP4 also plays a role in crossover formation, and its knockdown may reduce the frequency of chiasma formation that leads to sterility (Shen *et al.*, 2012). The study of Huang *et al.* (2024) not only highlights the molecular pathway of embryo sac development but also advocates for more detailed investigations of meiotic recombination and synopsis for a better understanding of the ZIP4–OsRH52A–OsMFS1 complex.

## *OsRH52A* affects the expression of downstream genes involved in megaspore development

To obtain a deeper understanding of downstream genes involved in megaspore development, a comparative transcriptome analysis of the WT and *rh52a* was carried out at meiosis and functional megaspore development stages. A total of 594 and 787 differentially expressed genes (DEGs) were up- and down-regulated at each developmental stage. Gene Ontology (GO) enrichment analysis indicated that the most enriched GO term was ‘microtubule-related processes’, which suggests that down-regulated DEGs were mainly responsible for defective embryo sac development. Notably, among the down-regulated DEGs, *MULTIPLE SPOROCYTE1* (*OsMSP1*) encodes a LEUCINE-RICH PROTEIN KINASE that controls the number of sporocytes in ovule development (Zhao *et al.*, 2008). Similarly, *HYBRID STERILITY-A1* (*HSA1*) encodes a protein of DOMAIN OF UNKNOWN FUNCTION (DUF1618) and controls the sterility of both F_1_ and F_2_ hybrids (Kubo *et al.*, 2016). Further studies on single and double mutants of *rh52a* with *hsa1* and *msp1* will help to understand why there is a sudden loss in the seed setting in F_2_ compared with F_1_, which remains open to study. In addition, it is thus far not known whether OsRH52A has the potential to be utilized for induction of synthetic apospory (development of an embryo from the nucellus without meiosis), which is considered an alternative mechanism to avoid heavy losses in F_2_ during hybrid seed production.
